# Recent advances in the management of nasopharyngeal carcinoma

**DOI:** 10.12688/f1000research.15066.1

**Published:** 2018-11-21

**Authors:** W. K. Jacky Lam, Jason Y. K. Chan

**Affiliations:** 1 Department of Chemical Pathology, Li Ka Shing Institute of Health Sciences, State Key Laboratory in Oncology in South China, The Chinese University of Hong Kong, Prince of Wales Hospital, Shatin, Hong Kong; 2 Department of Otorhinolaryngology, Head and Neck Surgery, The Chinese University of Hong Kong, Prince of Wales Hospital, Shatin, Hong Kong

**Keywords:** Nasopharyngeal carcinoma, EBV DNA, robotic surgery, endoscopic surgery, nasopharyngectomy, immunotherapy

## Abstract

Over the last few years, certain areas in the management nasopharyngeal carcinoma (NPC) that have an impact on the care of these patients have evolved, particularly with regard to liquid biopsies, minimally invasive surgery, and advances in chemotherapy and immunotherapy. Beyond its proven role in the diagnostics, surveillance, and treatment of NPC, liquid biopsy with plasma Epstein–Barr virus DNA in the screening of high-risk populations for NPC is strongly supported by recent evidence. Surgery of the nasopharynx is reserved for locally recurrent NPC, and in recent years there have been great strides in minimally invasive techniques with survival rates similar to those of open techniques in treating NPC. Induction chemotherapy in a recent pooled analysis was shown to be superior to concurrent chemotherapy alone for locoregionally advanced NPC. Finally, immunotherapy with a PD-1 inhibitor in NPC has been shown to have 1-year overall survival rates comparable to those of other patients with heavily pre-treated metastatic or recurrent NPC. In this commentary, we discuss these recent advances and their potential in the clinical management of patients with NPC.

## Introduction

Nasopharyngeal carcinoma (NPC) has a distinct geographical pattern of incidence. It is most prevalent in Southern China, where the annual incidence is about 30 cases per 100,000 persons ^1^, in contrast to fewer than 1 case per 100,000 persons in the US and Europe ^2^. NPC is associated with multiple risk factors, including Epstein–Barr virus (EBV) infection ^3^, genetic predisposition ^4^, and environmental factors ^5^. In particular, NPC associated with an undifferentiated carcinoma requires EBV for its development. Recently, there have been a number of advances in the management of NPC in screening, minimally invasive surgery, and immunotherapy that we are going to discuss in this review.

## Nasopharyngeal carcinoma screening and detection with plasma Epstein–Barr virus DNA

### Screening for nasopharyngeal carcinoma with plasma Epstein–Barr virus DNA

For almost all NPC cases in endemic regions, the tumor cells harbor the EBV genome ^6^. Because of the strong association with EBV, viral nucleic acids ^7^ or the host antibody response to the virus ^8, 9^ has been explored as a biomarker for NPC. Circulating EBV DNA in plasma as a cancer biomarker has been studied extensively for the monitoring and prognostication of NPC. It is used as an adjunct ^10^ to endoscopy and imaging for surveillance of recurrence after radical treatment. Pre-, mid-, and post-treatment levels of plasma EBV DNA ^11– 14^ have also been evaluated for their prognostication values in patients with NPC. Recently, a large-scale prospective study involving 20,000 asymptomatic male subjects in an endemic region confirmed the additional role of plasma EBV DNA for screening of NPC ^15^. Subjects who had any detectable levels of plasma EBV DNA by quantitative polymerase chain reaction (qPCR)-based assay on two consecutive occasions were defined as “screen positive”. “Screen-positive” subjects would subsequently undergo endoscopy and magnetic resonance imaging to confirm the diagnosis. The benefit of early detection was illustrated by a higher proportion of early NPC cases (stage I and II) among the screened cohort compared with an unscreened cohort. It was also shown that the patients with NPC detected by screening had a better progression-free survival (PFS) than did the same unscreened cohort. The promising results could provide a basis to further investigate population-wide adoption of a plasma EBV DNA-based screening program in endemic regions.

### Detection of primary and local persistent or recurrent nasopharyngeal carcinoma with a nasopharyngeal brush for Epstein–Barr virus DNA

Besides the detection of EBV DNA in plasma, researchers have shown that the detection of EBV DNA in nasopharyngeal brush cytology can be used for NPC detection at high sensitivity and specificity ^16^. Higher quantitative levels of EBV DNA by qPCR analysis were found in the nasopharyngeal brush cytology specimens of NPC patients than in non-NPC patients. The cytology specimens were obtained through a transoral route without endoscopic guidance; thus, use was not restricted to specialists. This may facilitate use in the community setting. The same brush system has been studied in another case control study and demonstrates the clinical potential for the detection of local recurrence in post-irradiated NPC patients ^17^. To further understand the role of the brush system in detecting locally persistent or recurrent disease, the same system is being trialed in sensitivity and specificity of a combination EBV DNA and methylation markers in both a nasopharyngeal brush and plasma (ClinicalTrials.gov Identifier: NCT03379610).

### Future perspectives for Epstein–Barr virus-associated biomarkers in nasopharyngeal carcinoma

There has been active research on EBV-associated NPC biomarkers. Further recent advances in EBV DNA detection in plasma have shown, through paired-end massively parallel sequencing, that size profile differences of EBV DNA identified amongst NPC and non-NPC patients may allow more specific identification of NPC ^18^. EBV antibody response has been widely studied for NPC risk prediction. A recent comprehensive evaluation of the EBV antibody repertoire (of both IgA and IgG responses) identified antibody targets—in addition to VCA and EBNA1 IgA biomarkers—which improved NPC risk stratification ^19^. Researchers have also explored the viral messenger RNAs and methylation status of the C-promoter region of EBV in cytology specimens as NPC biomarkers ^20^. In addition, there are explorations of microRNAs in plasma as potential biomarkers ^21, 22^. Further study is required to understand the clinical values of these additional analyses.

## Surgery in nasopharyngeal carcinoma

Nasopharyngectomy is one established treatment option for locally recurrent NPC. The conventional open approaches for nasopharyngectomy include the maxillary swing, midface degloving, transpalatal, transmaxillary, and trans-infratemporal fossa approaches ^23– 25^. In the last few years, there have been advancements in minimally invasive techniques. Endoscopic nasopharyngectomy was first used for the resection of early stage recurrences ^26, 27^. With the advancements in endoscopy technologies (including optics and navigation ^28^) and accumulation of experience, endoscopic nasopharyngectomy is no longer used for small NPC recurrences only. More extensive endoscopic resection is now feasible for the more advanced recurrences, including rT3 and rT4 diseases ^29^. The recurrent tumor is considered inoperable only when it has substantial intracranial extension with cavernous sinus invasion or encasement of the petrosal internal carotid artery (ICA). A retrospective review including selected rT1–rT3 patients in a single center showed a better overall survival (OS) for the endoscopic surgery group than the re-irradiation group ^30^.

An alternative minimally invasive approach is the use of robotics for nasopharyngectomy. Tsang *et al*. used this approach with the da Vinci S system (Intuitive Surgical Inc., Sunnyvale, CA, USA) and reported their early results: a 2-year OS and a disease-free survival of 83% and 61%, respectively ^31^. However, this approach is hampered by the need for splitting the palate in an irradiated field and the lack of bone drills to address the extensive bony landmarks in the nasopharynx. Further advancements with novel flexible robotic systems aim to overcome some of these limitations. With the Flex ^®^ system (Medrobotics, Raynham, MA, USA), a highly articulate endoscope with flexible instrumentation, a transoral palate-sparing approach to the nasopharynx for a nasopharyngectomy was shown to be feasible in preclinical studies ^32^. Similarly, with the da Vinci SP system (Intuitive Surgical Inc.), a palate-sparing approach could be used to resect the nasopharynx en bloc in preclinical studies ^33^. Finally, a recent clinical trial of the da Vinci SP system also showed that the nasopharynx can be approached without splitting the palate; however, this has not confirmed the feasibility of resecting the nasopharynx ^34^.

On the other hand, for advanced rT3 and rT4 recurrences, resection of the tumor via a combined craniofacial resection has been proposed ^35^. Staged extracranial/intracranial vascular bypass was first performed prior to the combined craniofacial resection to secure the cerebral blood flow. The surgical outcomes were reported in a single-center study involving 28 patients with rT3 or rT4 diseases. The 5-year OS was reported to be 52%, and 13 patients achieved a microscopically clear resection margin. Despite these survival rates, there was a significant deterioration in physical functioning scores affecting speech and swallowing, resulting in a significant effect on patients’ quality of life.

Overall, these advancements in minimally invasive options and the development of more approaches for locally advanced recurrent NPC provide better options with the prospect of improving survival and quality of life for these patients with locally recurrent NPC. In the future, with the detection of NPC at earlier stages, these minimally invasive approaches may provide an opportunity to explore the role of primary surgery in NPC, similar to the treatment of another virally mediated head and neck cancer (human papilloma virus-positive oropharyngeal carcinoma).

## Chemotherapy and immune checkpoint inhibitors

There have been recent advances in the understanding of the role of induction chemotherapy for locoregionally advanced NPC. Induction chemotherapy with concurrent chemotherapy versus concurrent chemotherapy alone has been primarily evaluated with varying results in four recent randomized control trials in locoregionally advanced NPC in Hong Kong, Singapore, and Guangzhou. A pooled analysis of these studies that included 1,193 patients with no heterogeneity showed significant improvements in PFS and OS for patients in the induction chemotherapy arm ^36^. As a result of these findings, the National Comprehensive Cancer Network guidelines upgraded the evidence of induction chemotherapy to level 2A for locoregionally advanced NPC.

Cancer immunotherapy through the blockade of immune checkpoint has revolutionized the management of advanced-stage cancers, including metastasis and recurrence. Promising results of the use of the anti-CTLA-4 antibody ipilimumab in advanced melanoma cases have driven interest in exploring the use of immune checkpoint inhibitors in other cancers. There are a number of clinical trials to evaluate these therapies in different types of cancer, and the anti-tumor activities in different cancers were shown to be variable. The clinical efficacy of one immune checkpoint inhibitor, the anti-PD-1 antibody nivolumab, for heavily pretreated recurrent or metastatic NPC was recently evaluated in a multinational study ^37^. For the 44 NPC patients in the study, the overall objective response rate was reported to be 20.5%. The response to nivolumab was not statistically associated with PD-L1 expression in tumor cells or immune cells in the archived tumor samples, but such expression was shown to be predictive of the response to PD-1 inhibitors in lung cancer ^38^. Of note, the expression of HLA-A and HLA-B genes, though not predictive of the response to nivolumab, was found to be associated with PFS. Patients with NPC which had loss of HLA-A or HLA-B expression (or both) had better PFS. This is in contrast to the other study findings that loss of HLA class 1 expression is associated with poorer prognosis in other solid cancers.

Ongoing trials are investigating the role of immunotherapy in the treatment of NPC in the primary setting as a neoadjuvant, concurrent, and adjuvant therapy that will further define its role. Nivolumab and ipilimumab (CTLA-4 inhibitor) as a combinatorial therapy are being evaluated in rare tumors that include NPC (ClinicalTrials.gov Identifier: NCT02834013), and nivolumab with chemoradiation in advanced-stage NPC is being evaluated in a phase 2 clinical trial (ClinicalTrials.gov Identifier: NCT03267498) and in a randomized phase 3 trial of adjuvant PD-1 antibody or observation in patients with locoregional advanced NPC (ClinicalTrials.gov Identifier: NCT03427827).

## Conclusions

There has been substantial advancement in the diagnostics and treatment, both surgical and non-surgical, for NPC. Plasma EBV DNA has been shown to be a powerful biomarker in the screening, diagnosing, surveillance, and treatment of NPC. The repertoire of minimally invasive approaches and opportunities for nasopharyngectomy is improving at a rapid pace with less morbidity for locally recurrent NPC. Finally, advances in therapeutics, namely immunotherapy, are just beginning to be investigated. With all of these exciting recent advances, we are looking forward to future studies which further improve our understanding of NPC and significantly improve the current management of these patients.
